# Bis(4-hy­droxy­benzoato-κ^2^ *O*,*O*′)bis­(pyridine-κ*N*)copper(II)

**DOI:** 10.1107/S1600536811023038

**Published:** 2011-06-18

**Authors:** Lailatun Nazirah Ozair, Norbani Abdullah, Kong Mun Lo

**Affiliations:** a Department of Chemistry, University of Malaya, 50603 Kuala Lumpur, Malaysia

## Abstract

In the title compound, [Cu(C_7_H_5_O_3_)_2_(C_5_H_5_N)_2_], the Cu atom is located on an inversion center and is coordinated by the N atoms of the two pyridine ligands, *trans* to each other, and to the carboxyl­ate O atoms of two bidentate 4-hy­droxy­benzoate ligands [Cu—O = 1.9706 (10) and 2.5204 (11) Å]. Hydrogen bonding between hy­droxy H and carboxyl­ate O atoms results in a layer structure parallel to the *ab* plane.

## Related literature

For the structure of bis­(*p*-hy­droxy­benzoate)dipicoline–copper(II), see: Sharma *et al.* (2009 ▶).
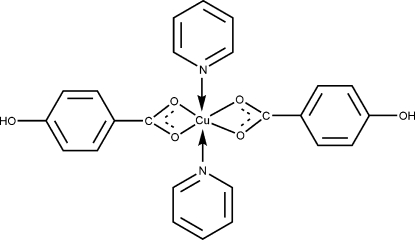


## Experimental

### 

#### Crystal data

[Cu(C_7_H_5_O_3_)_2_(C_5_H_5_N)_2_]
*M* *_r_* = 495.96
Monoclinic, 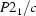

*a* = 10.6715 (2) Å
*b* = 8.5385 (1) Å
*c* = 12.3988 (2) Å
β = 109.124 (1)°
*V* = 1067.41 (3) Å^3^
*Z* = 2
Mo *K*α radiation
μ = 1.07 mm^−1^
*T* = 100 K
0.30 × 0.26 × 0.20 mm

#### Data collection

Bruker SMART APEXII diffractometer
Absorption correction: multi-scan (*SADABS*; Sheldrick, 1996 ▶) *T*_min_ = 0.663, *T*_max_ = 0.746
9756 measured reflections
2448 independent reflections
2202 reflections with *I* > 2σ(*I*)
*R*_int_ = 0.023

#### Refinement

*R*[*F* ^2^ > 2σ(*F* ^2^)] = 0.026
*wR*(*F* ^2^) = 0.066
*S* = 1.06
2448 reflections
152 parameters
H-atom parameters constrained
Δρ_max_ = 0.38 e Å^−3^
Δρ_min_ = −0.32 e Å^−3^

### 

Data collection: *APEX2* (Bruker, 2008 ▶); cell refinement: *SAINT* (Bruker, 2008) ▶; data reduction: *SAINT* ▶; program(s) used to solve structure: *SHELXS97* (Sheldrick, 2008 ▶); program(s) used to refine structure: *SHELXL97* (Sheldrick, 2008 ▶); molecular graphics: *X-SEED* (Barbour, 2001 ▶); software used to prepare material for publication: *publCIF* (Westrip, 2010 ▶).

## Supplementary Material

Crystal structure: contains datablock(s) I, global. DOI: 10.1107/S1600536811023038/om2435sup1.cif

Structure factors: contains datablock(s) I. DOI: 10.1107/S1600536811023038/om2435Isup2.hkl

Additional supplementary materials:  crystallographic information; 3D view; checkCIF report

## Figures and Tables

**Table 1:** Hydrogen-bond geometry (Å, °)

*D*—H⋯*A*	*D*—H	H⋯*A*	*D*⋯*A*	*D*—H⋯*A*
O3—H3*A*⋯O2^i^	0.84	1.87	2.7028 (16)	171

Symmetry code: (i) 
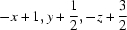
.

**Fig. 1.**
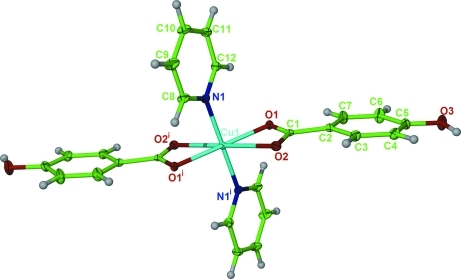
The molecular structure of bis(4-hydroxybenzoato-O, O')dipyridylcopper(II), showing 50% probability displacement ellipsoids and the atom numbering. Hydrogen atoms are drawn as spheres of arbitrary radius. (Symmetry code (i): -x + 2, -y, -z + 2).

**Fig. 2.**
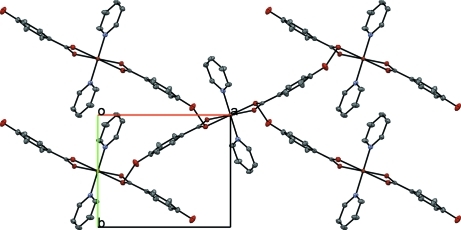
A view down the c-axis of the crystal packing of the title compound. Hydrogen atoms have been omitted for clarity and the O—H···O hydrogen bonds are shown as red dotted lines.

## supplementary crystallographic information

### Comment

The copper atom in the title complex is located on an inversion center and adopts a distorted octahedral geometry, with the oxygen atoms of the carboxylic groups occupying the equatorial positions (Fig. 1). The axial Cu—N bond distance of 2.0076 (13) Å is comparable with that of bis(*p*-hydroxybenzoate)dipicoline-copper(II) which is 1.987 (2) Å (Sharma *et al.*, 2009). The 4-hydroxybenzoate group acts as a bidentate ligand with Cu—O bond distances of 1.9706 (10) and 2.5204 (11) Å. The dipicoline-copper(II) complex differs from the title complex in that the two picoline ligands are *cis* to each other (N—Cu—N 91.50 (10)*^o^*) whereas the two pyridine ligands in the title complex are *trans* to each other. The distortion from ideal octahedral geometry for the title compound is mainly due to the small bite angle (57.60 (4)°) formed by the bidentate carboxylate moiety. In the crystal structure, intermolecular O—H···O hydrogen bonds link the molecules into layers parallel to the *ab* plane (Fig. 2). The π-π contacts between the pyridine rings, *Cg*1-*Cg*1' [symmetry code: 2 - *x*, 1 - *y*, 2 - *z*, where *Cg*1 is the centroid of the ring (N1, C8—C12)] may further stabilize the overall structure, with centroid-centroid distance of 3.7878 (10) Å.

### Experimental

*p*-Hydroxybenzoic acid (0.35 g, 2.5 mmol) was dissolved in 100 ml of ethanol. While stirring and gently heating the solution, copper(II) acetate monohydrate (0.26 g, 1.3 mmol) was added portionwise. This was followed by 0.5 ml of pyridine and the mixture was heated for 30 minutes. The solution mixture was then filtered and upon cooling of the filtrate gave the title compound as a dark green crystalline solid.

### Refinement

Hydrogen atoms were placed at calculated positions (C–H 0.95 Å) and were treated as riding on their parent carbon atoms, with *U*(H) set to 1.2–1.5 times *U*\~eq\~(C). The hydroxy-H was refined with a restraint of 0.84 ± 0.01 Å.

### Crystal data

**Table:**

[Cu(C_7_H_5_O_3_)_2_(C_5_H_5_N)_2_]	*F*(000) = 510
*M**_r_* = 495.96	*D*_x_ = 1.543 Mg m^−^^3^
Monoclinic, *P*2_1_/*c*	Mo *K*α radiation, λ = 0.71073 Å
Hall symbol: -P 2ybc	Cell parameters from 4633 reflections
*a* = 10.6715 (2) Å	θ = 3.0–28.2°
*b* = 8.5385 (1) Å	µ = 1.07 mm^−^^1^
*c* = 12.3988 (2) Å	*T* = 100 K
β = 109.124 (1)°	Block, dark green
*V* = 1067.41 (3) Å^3^	0.30 × 0.26 × 0.20 mm
*Z* = 2	

### Data collection

**Table:**

Bruker SMART APEXII diffractometer	2448 independent reflections
Radiation source: fine-focus sealed tube	2202 reflections with *I* > 2σ(*I*)
graphite	*R*_int_ = 0.023
φ and ω scans	θ_max_ = 27.5°, θ_min_ = 2.0°
Absorption correction: multi-scan (*SADABS*; Sheldrick, 1996)	*h* = −13→13
*T*_min_ = 0.663, *T*_max_ = 0.746	*k* = −11→11
9756 measured reflections	*l* = −16→16

### Refinement

**Table:**

Refinement on *F*^2^	Primary atom site location: structure-invariant direct methods
Least-squares matrix: full	Secondary atom site location: difference Fourier map
*R*[*F*^2^ > 2σ(*F*^2^)] = 0.026	Hydrogen site location: inferred from neighbouring sites
*wR*(*F*^2^) = 0.066	H-atom parameters constrained
*S* = 1.06	*w* = 1/[σ^2^(*F*_o_^2^) + (0.0301*P*)^2^ + 0.7197*P*] where *P* = (*F*_o_^2^ + 2*F*_c_^2^)/3
2448 reflections	(Δ/σ)_max_ < 0.001
152 parameters	Δρ_max_ = 0.38 e Å^−^^3^
0 restraints	Δρ_min_ = −0.32 e Å^−^^3^

### Special details

**Table:**

Geometry. All e.s.d.'s (except the e.s.d. in the dihedral angle between two l.s. planes) are estimated using the full covariance matrix. The cell e.s.d.'s are taken into account individually in the estimation of e.s.d.'s in distances, angles and torsion angles; correlations between e.s.d.'s in cell parameters are only used when they are defined by crystal symmetry. An approximate (isotropic) treatment of cell e.s.d.'s is used for estimating e.s.d.'s involving l.s. planes.
Refinement. Refinement of *F*^2^ against ALL reflections. The weighted *R*-factor *wR* and goodness of fit *S* are based on *F*^2^, conventional *R*-factors *R* are based on *F*, with *F* set to zero for negative *F*^2^. The threshold expression of *F*^2^ > σ(*F*^2^) is used only for calculating *R*-factors(gt) *etc*. and is not relevant to the choice of reflections for refinement. *R*-factors based on *F*^2^ are statistically about twice as large as those based on *F*, and *R*- factors based on ALL data will be even larger.

### Fractional atomic coordinates and isotropic or equivalent isotropic displacement parameters (Å^2^)

**Table:**

	*x*	*y*	*z*	*U*_iso_*/*U*_eq_	
Cu1	1.0000	0.0000	1.0000	0.01147 (8)	
O1	0.82299 (10)	0.04277 (13)	1.01071 (9)	0.0143 (2)	
O2	0.81152 (11)	0.10333 (13)	0.83387 (9)	0.0162 (2)	
O3	0.28650 (12)	0.42997 (16)	0.85782 (10)	0.0258 (3)	
H3A	0.2618	0.4788	0.7956	0.039*	
N1	1.05579 (12)	0.21481 (15)	1.06344 (11)	0.0139 (3)	
C1	0.76211 (15)	0.10384 (17)	0.91341 (13)	0.0138 (3)	
C2	0.63226 (15)	0.18074 (17)	0.89663 (13)	0.0152 (3)	
C3	0.56256 (16)	0.24646 (19)	0.79114 (14)	0.0181 (3)	
H3	0.5956	0.2350	0.7291	0.022*	
C4	0.44571 (16)	0.32832 (19)	0.77537 (14)	0.0191 (3)	
H4	0.3985	0.3717	0.7028	0.023*	
C5	0.39815 (16)	0.3465 (2)	0.86627 (14)	0.0193 (3)	
C6	0.46532 (18)	0.2783 (2)	0.97123 (15)	0.0270 (4)	
H6	0.4314	0.2881	1.0328	0.032*	
C7	0.58159 (17)	0.1960 (2)	0.98591 (14)	0.0232 (4)	
H7	0.6271	0.1496	1.0577	0.028*	
C8	1.01481 (17)	0.27077 (19)	1.14781 (14)	0.0188 (3)	
H8	0.9540	0.2105	1.1719	0.023*	
C9	1.05787 (19)	0.4126 (2)	1.20074 (15)	0.0242 (4)	
H9	1.0285	0.4478	1.2611	0.029*	
C10	1.14449 (18)	0.50256 (19)	1.16446 (15)	0.0226 (3)	
H10	1.1758	0.6003	1.1996	0.027*	
C11	1.18455 (16)	0.4474 (2)	1.07611 (15)	0.0205 (3)	
H11	1.2425	0.5076	1.0485	0.025*	
C12	1.13899 (15)	0.30311 (18)	1.02847 (14)	0.0170 (3)	
H12	1.1678	0.2651	0.9685	0.020*	

### Atomic displacement parameters (Å^2^)

**Table:**

	*U*^11^	*U*^22^	*U*^33^	*U*^12^	*U*^13^	*U*^23^
Cu1	0.01118 (13)	0.00913 (13)	0.01483 (14)	−0.00004 (9)	0.00523 (9)	−0.00102 (10)
O1	0.0129 (5)	0.0132 (5)	0.0172 (5)	0.0019 (4)	0.0054 (4)	0.0005 (4)
O2	0.0161 (5)	0.0159 (5)	0.0175 (5)	−0.0010 (4)	0.0066 (4)	−0.0009 (4)
O3	0.0220 (6)	0.0344 (7)	0.0245 (6)	0.0126 (5)	0.0124 (5)	0.0100 (5)
N1	0.0142 (6)	0.0115 (6)	0.0158 (6)	0.0012 (5)	0.0045 (5)	0.0001 (5)
C1	0.0132 (7)	0.0085 (6)	0.0193 (7)	−0.0027 (5)	0.0047 (6)	−0.0019 (6)
C2	0.0128 (7)	0.0117 (7)	0.0200 (8)	−0.0013 (6)	0.0039 (6)	−0.0020 (6)
C3	0.0199 (8)	0.0170 (7)	0.0186 (8)	0.0011 (6)	0.0080 (6)	−0.0001 (6)
C4	0.0204 (8)	0.0188 (8)	0.0173 (7)	0.0026 (6)	0.0050 (6)	0.0027 (6)
C5	0.0140 (7)	0.0208 (8)	0.0237 (8)	0.0024 (6)	0.0070 (6)	0.0037 (7)
C6	0.0255 (9)	0.0378 (11)	0.0227 (9)	0.0111 (8)	0.0148 (7)	0.0085 (8)
C7	0.0207 (8)	0.0299 (9)	0.0197 (8)	0.0066 (7)	0.0076 (6)	0.0075 (7)
C8	0.0265 (8)	0.0140 (7)	0.0199 (8)	−0.0026 (6)	0.0130 (7)	−0.0004 (6)
C9	0.0374 (10)	0.0175 (8)	0.0222 (8)	−0.0034 (7)	0.0160 (7)	−0.0052 (7)
C10	0.0292 (9)	0.0139 (7)	0.0251 (8)	−0.0052 (7)	0.0094 (7)	−0.0049 (7)
C11	0.0202 (8)	0.0151 (7)	0.0281 (9)	−0.0039 (6)	0.0107 (7)	−0.0009 (7)
C12	0.0169 (7)	0.0151 (7)	0.0210 (8)	−0.0003 (6)	0.0089 (6)	−0.0016 (6)

### Geometric parameters (Å, °)

**Table:**

Cu1—O1	1.9706 (10)	C4—H4	0.9500
Cu1—N1	2.0076 (13)	C5—C6	1.391 (2)
Cu1—O2	2.5204 (11)	C6—C7	1.385 (2)
O1—C1	1.2790 (19)	C6—H6	0.9500
O2—C1	1.2614 (18)	C7—H7	0.9500
O3—C5	1.3624 (19)	C8—C9	1.382 (2)
O3—H3A	0.8400	C8—H8	0.9500
N1—C12	1.340 (2)	C9—C10	1.386 (2)
N1—C8	1.347 (2)	C9—H9	0.9500
C1—C2	1.485 (2)	C10—C11	1.383 (2)
C2—C7	1.388 (2)	C10—H10	0.9500
C2—C3	1.393 (2)	C11—C12	1.384 (2)
C3—C4	1.386 (2)	C11—H11	0.9500
C3—H3	0.9500	C12—H12	0.9500
C4—C5	1.388 (2)		
			
O1—Cu1—O1^i^	180.0	C5—C4—H4	120.2
O1—Cu1—N1^i^	91.60 (5)	O3—C5—C4	122.64 (15)
O1—Cu1—N1	88.40 (5)	O3—C5—C6	117.43 (15)
O1^i^—Cu1—N1	91.60 (5)	C4—C5—C6	119.94 (15)
N1^i^—Cu1—N1	180.0	C7—C6—C5	120.02 (15)
O1—Cu1—O2	57.60 (4)	C7—C6—H6	120.0
O1^i^—Cu1—O2	122.40 (4)	C5—C6—H6	120.0
N1^i^—Cu1—O2	86.83 (4)	C6—C7—C2	120.61 (15)
N1—Cu1—O2	93.17 (4)	C6—C7—H7	119.7
C1—O1—Cu1	102.35 (9)	C2—C7—H7	119.7
C1—O2—Cu1	77.78 (8)	N1—C8—C9	122.52 (15)
C5—O3—H3A	109.5	N1—C8—H8	118.7
C12—N1—C8	118.01 (13)	C9—C8—H8	118.7
C12—N1—Cu1	122.01 (10)	C8—C9—C10	119.02 (15)
C8—N1—Cu1	119.89 (10)	C8—C9—H9	120.5
O2—C1—O1	121.52 (14)	C10—C9—H9	120.5
O2—C1—C2	120.11 (14)	C11—C10—C9	118.72 (15)
O1—C1—C2	118.34 (13)	C11—C10—H10	120.6
C7—C2—C3	118.88 (14)	C9—C10—H10	120.6
C7—C2—C1	121.28 (14)	C10—C11—C12	119.01 (15)
C3—C2—C1	119.75 (14)	C10—C11—H11	120.5
C4—C3—C2	120.95 (15)	C12—C11—H11	120.5
C4—C3—H3	119.5	N1—C12—C11	122.69 (15)
C2—C3—H3	119.5	N1—C12—H12	118.7
C3—C4—C5	119.55 (15)	C11—C12—H12	118.7
C3—C4—H4	120.2		
			
O1^i^—Cu1—O1—C1	−98 (46)	O1—C1—C2—C7	4.3 (2)
N1^i^—Cu1—O1—C1	−90.19 (9)	O2—C1—C2—C3	2.7 (2)
N1—Cu1—O1—C1	89.81 (9)	O1—C1—C2—C3	−179.10 (14)
O2—Cu1—O1—C1	−4.95 (8)	C7—C2—C3—C4	1.1 (2)
O1—Cu1—O2—C1	5.02 (8)	C1—C2—C3—C4	−175.51 (14)
O1^i^—Cu1—O2—C1	−174.98 (8)	C2—C3—C4—C5	0.7 (2)
N1^i^—Cu1—O2—C1	98.93 (9)	C3—C4—C5—O3	177.74 (15)
N1—Cu1—O2—C1	−81.07 (9)	C3—C4—C5—C6	−2.2 (3)
O1—Cu1—N1—C12	−143.73 (12)	O3—C5—C6—C7	−178.07 (17)
O1^i^—Cu1—N1—C12	36.27 (12)	C4—C5—C6—C7	1.9 (3)
N1^i^—Cu1—N1—C12	0(100)	C5—C6—C7—C2	0.0 (3)
O2—Cu1—N1—C12	−86.30 (12)	C3—C2—C7—C6	−1.5 (3)
O1—Cu1—N1—C8	39.72 (12)	C1—C2—C7—C6	175.12 (17)
O1^i^—Cu1—N1—C8	−140.28 (12)	C12—N1—C8—C9	−1.7 (2)
N1^i^—Cu1—N1—C8	0(95)	Cu1—N1—C8—C9	175.01 (13)
O2—Cu1—N1—C8	97.15 (12)	N1—C8—C9—C10	1.3 (3)
Cu1—O2—C1—O1	−7.68 (13)	C8—C9—C10—C11	0.2 (3)
Cu1—O2—C1—C2	170.43 (13)	C9—C10—C11—C12	−1.2 (3)
Cu1—O1—C1—O2	9.84 (16)	C8—N1—C12—C11	0.6 (2)
Cu1—O1—C1—C2	−168.30 (11)	Cu1—N1—C12—C11	−176.06 (12)
O2—C1—C2—C7	−173.84 (15)	C10—C11—C12—N1	0.9 (3)

Symmetry codes: (i) −*x*+2, −*y*, −*z*+2.

### Hydrogen-bond geometry (Å, °)

**Table:**

*D*—H···*A*	*D*—H	H···*A*	*D*···*A*	*D*—H···*A*
O3—H3A···O2^ii^	0.84	1.87	2.7028 (16)	171

Symmetry codes: (ii) −*x*+1, *y*+1/2, −*z*+3/2.
